# Comparative study of virulence potential, phylogenetic origin, CRISPR-*Cas* regions and drug resistance of *Escherichia coli* isolates from urine and other clinical materials

**DOI:** 10.3389/fmicb.2023.1289683

**Published:** 2023-11-29

**Authors:** Anna Dziuba, Sylwia Dzierżak, Anna Sodo, Monika Wawszczak-Kasza, Katarzyna Zegadło, Jakub Białek, Natalia Zych, Wojciech Kiebzak, Jarosław Matykiewicz, Stanisław Głuszek, Wioletta Adamus-Białek

**Affiliations:** ^1^Institute of Medical Sciences, Jan Kochanowski University, Kielce, Poland; ^2^Department of Microbiology, Regional Hospital, Kielce, Poland; ^3^Institute of Health Science, Jan Kochanowski University, Kielce, Poland

**Keywords:** virulence-associated genes, drug resistance, *Escherichia coli*, CRISPR, phylogenetic

## Abstract

**Introduction:**

Urinary tract infections (UTI), among which the main etiological factor is uropathogenic *Escherichia coli* (UPEC, *E. coli*), remain an important issue for clinicians. The aim of the study was to demonstrate clear differences in the pathogenic properties of urine-derived *E. coli* compared to other extraintestinal *E. coli* clinical isolates (derived from: blood, lower respiratory tracts, sputum, reproductive tract, body fluids, perianal pus, other pus, wound, postoperative wound and other sources).

**Methods:**

The collection of 784 *E. coli* isolates was collected from various materials of hospitalized patients. They were analyzed in terms of virulence-associated genes (*papC, sfaD/sfaE, cnf1, usp., fimG/H, hlyA*), belonging to phylogenetic groups and the presence of CRISPR-*Cas* regions using PCR. In addition, the epidemiological data and the antibiotic resistance profiles provided by the hospital’s microbiology department were included for statistical analyses.

**Results:**

Urine-derived *E. coli* showed significantly greater virulence potential compared to other isolates, but they were generally unremarkable in terms of drug resistance. The isolates most often belonged to phylogenetic group B2. Drug resistance was negatively correlated with CRISPR 2 presence and high average virulence score, but positively correlated with CRISPR 4 presence. To the best of our knowledge, we are the first to report significant differences in sputum-derived isolates—they revealed the lowest virulence potential and, at the same time, the highest drug resistance.

**Discussion:**

In conclusion, we demonstrated significant differences of urinary-derived *E. coli* compared to other clinical *E. coli* isolates. We would like to suggest excluding penicillins from use in *E. coli* infection at this time and monitoring strains with a high pathogenicity potential.

## 1 Introduction

*Escherichia coli* is widely distributed around the world, and can be found both in water and soil. It is also part of the intestinal microbiota of animals and humans (Jang et al., 2017). This species, due to the acquisition of virulence-associated genes (VAGs), includes not only commensal strains, but also pathogenic ones (Russo and Johnson, 2000; Mainil, 2013; Kobayashi et al., 2021). Such a large variety of populated niches is due to the extraordinary plasticity of their genome. *E. coli* strains are known for their remarkable adaptabilities, which allows them to survive in changing and unfavorable environmental conditions (Kaper et al., 2004; Gomes et al., 2016). Therefore, *E. coli* is one of the monitored species in the context of alarmingly increasing drug resistance. In 2020, more than half of *E. coli* isolates were resistant to at least one antimicrobial group (European Centre for Disease Prevention and Control, 2022), making it the most reported bacteria in 29 European Union (EU) and European Economic Area (EEA) countries. There is a long history of epidemiological research on VAGs and the source of isolation distinguished pathogenic strains into intestinal *E. coli* (IPEC) and extraintestinal pathogenic *E. coli* (ExPEC). The ExPEC group includes the following strains: uropathogenic (UPEC), neonatal meningitis (NMEC), associated with the occurrence of sepsis (SEPEC) and avian (APEC) (Robins-Browne et al., 2016; Sarowska et al., 2019; Jesser and Levy, 2020; Riley, 2020). So far, it has not been possible to fully explain the processes responsible for the spread of ExPEC, therefore this topic requires further research. Similarly, in the case of uropathogenic strains, the literature indicates the intracellular pathogenicity characteristic of this pathotype, however clinicians usually refer to each strain isolated from the urine of a patient with urinary tract infection as UPEC. Generally, these strains are characterized by high variability of VAGs, most often those specific for UPEC encode adhesins (*fim*, *pap*, *sfa*, *Afa/Dr*, *focG*), α-hemolysin (*hly*), cytotoxic necrotizing factor type 1 (*cnf*1), adherence factor (*iha*), aerobactin receptor (*iutA*), yersiniabactin receptor (*fyuA*) and bacteriocin named as uropathogenic specific protein (*usp*) (Adamus-Bialek et al., 2009; Krawczyk et al., 2015; Paniagua-Contreras et al., 2018; Sarowska et al., 2019). Undoubtedly, they have a mechanism that is regulating drug resistance and virulence at the same time.

It has been observed that the strains are capable of permanent changes in pathogenicity (loss of pathogenicity factors, changes in biofilm formation) during the acquisition of resistance to antibiotics, mainly fluoroquinolones and beta-lactams (Harwalkar et al., 2014; Adamus-Białek et al., 2019). This is extremely important from the increasing drug resistance point of view—among uropathogenic strains the number of isolated MDR (multi-drug resistance) strains increases significantly (Cerceo et al., 2016) and they become less visible to the host by loss of antigens. The transmission ability of VAGs among *E. coli* strains is particularly important in the context of chronic infections and widespread antibiotic resistance, as well as the possibility of the emergence of new pathotypes with complex virulence. This mechanism is not fully understood. Recently, scientists have focused on the role of the CRISPR-*Cas* mechanism as a type of acquired immune response against bacteriophages (Samai et al., 2015; Whitaker and Vanderpool, 2016) results in an increased genome modification ability. The studies conducted so far indicate a relationship between the presence of the CRISPR sequence and decreased resistance to antibiotics, which leads to the conclusion that the presence of CRISPR limits the adaptability of the microorganism (García-Gutiérrez et al., 2015; Shehreen et al., 2019). Furthermore, studies have shown that some CRISPR are complementary to plasmids carrying resistance to selected antibiotics. Other studies have shown a relationship between an increased number of VAGs and fewer repeat sequences at the *E. coli* CRISPR loci (Heidelberg et al., 2009; Dang et al., 2013). Understanding the complex mechanisms of pathogenicity and antibiotic resistance of UPEC strains is an opportunity to develop new and improved therapies with social, environmental, and economic benefits. In this work, we present the comprehensive analysis of uropathogenic *E. coli* isolates against the background of *E. coli* isolated from other clinical sources. The collection of 784 *E. coli* isolates was examined in terms of selected VAGs, phylogenetic affiliation, CRISPR regions, as well as the drug resistance profiles. We wanted to verify to what extent selected pathogenic factors long recognized as typical for UPEC are widespread among various strains of *E. coli* and whether their presence correlates with other properties.

## 2 Materials and methods

### 2.1 *Escherichia coli* collection

A total of 784 *E. coli* isolates were donated for research by the Department of Microbiology, Regional Hospital in Kielce along with anonymous documentation regarding the source of isolation, drug resistance, as well as the patient’s sex and age.

The species identification was carried out in accordance with the diagnostic procedures according to quality control certificate (PN-EN 12322, PN-EN ISO 11133). The biological samples were incubated on Columbia agar with 5% blood cells (BioMaxima S.A.) and MacConkey agar (BioMaxima S.A.) in aerobic conditions and at a temperature 35–37°C for 18–24 h. The species affiliation was determined automatically by incubating single bacterial colony into VITEK 2 apparatus (BioMerieux) using ID Cards designed to identify Gram-negative rods at the species level (GN card). The microorganism identification system complies with the requirements of ISO 13485 and FDA Quality System Regulation (QSR) in terms of design, development, and implementation. The bacteria isolated from urine were identified as *E. coli* by CHROMagar Orientation Differential Medium (GRASO), 99.3% specificity for *E. coli* (pink colonies).

Antibiotic susceptibility testing of identified *E. coli* was carried out via VITEK 2 apparatus (BioMerieux) using AST Cards for the determination of antibiotic susceptibility of gram-negative *E. coli*: AST-N330 [ampicillin, amoxicillin/clavulanic acid, cephalexin, cefuroxime, nitrofurantoin, norfloxacin, ciprofloxacin, trimethoprim-sulfamethoxazole, amikacin, gentamicin, piperacillin-tazobactam, ceftazidime, meropenem, cefotaxime (also representative of ceftriaxone), ertapenem as the most sensitive indicator of emerging resistance to carbapenems], AST-N332 (amoxicillin/clavulanic acid, cefuroxime, cefotaxime, ciprofloxacin, trimethoprim-sulfamethoxazole, amikacin, gentamicin, tobramycin, piperacillin-tazobactam, ceftazidime, meropenem, imipenem, cefotaxime, and advanced expert system showing the exact phenotypic profile along with the resistance mechanisms). The ranges of antibiotic concentrations in the cards comply with the applicable EUCAST recommendations.

The disc diffusion method was used to detect resistance mechanisms of producing ESBL (extended-spectrum beta-lactamases), AmpC (AmpC beta-lactamases), KPC (*Klebsiella pneumoniae* carbapenemases), MBL (metallo-beta-lactamases), OXA 48 (oxacillinase-48) and to determine the drug susceptibility of drugs supplementing the cards, in accordance with the EUCAST recommendations [version 7.0–11.0]. The antibiograms were performed on Mueller–Hinton II 2 LAB-AGAR (Biomaxima, PN-EN 12322) using commercial disks (Oxoid, Wesel, Germany). Bacterial isolates were determined as sensitive (S), intermediately sensitive (I) or resistant (R) to the antibiotics.

The bacterial isolates were collected from inpatients in the period from 2017 to 2022 (Table 1) and were stored in a 50% glycerin solution at −80°C in the Laboratory of Medical Genetics, at the Institute of Medical Sciences, Jan Kochanowski University in Kielce, Poland.

**Table 1:** The clinical characteristic of studied *E. coli* isolates.

No.	Source	Total (*n*)	Male *n* (%)	Female *n* (%)	*p*-value	Age (Q1; Q3)	Age_M_ (Q1; Q3)	Age_F_ (Q1; Q3)	*p*-value
1	Urine	197	61 (31)	136 (69)	<0.001 (χ^2^)	21 (0; 72)	14 (0; 66)	23 (0; 75)	ns (†)
2	Blood	128	62 (48)	66 (52)	ns (χ^2^)	70 (59; 80)	67 (56; 77)	73 (61; 82)	ns (†)
3	LRT	85	60 (71)	25 (34)	<0.001 (χ^2^)	63 (51; 72)	63 (52; 69)	62 (45; 78)	ns (†)
4	Sputum	20	13 (65)	7 (35)	ns (χ^2^)	80 (65; 84)	75 (62; 86)	83 (74; 84)	ns (#)
5	FRT	26	na	26 (100)	na	38.5 (31; 44)	na	38.5 (31; 44)	na
6	Wound	73	43 (59)	30 (41)	ns (χ^2^)	71.5 (48; 77)	60 (37; 69)	71.5 (58; 78)	<0.01 (†)
7	POW	24	8 (33)	16 (67)	0.090 (χ^2^)	69 (55; 80)	69 (62; 81)	67.5 (55; 80)	ns (†)
8	BF	160	95 (59)	65 (41)	<0.05 (χ^2^)	15 (9; 50)	13 (9; 34)	19 (11; 60)	<0.01 (†)
9	Perianal pus	26	20 (77)	6 (23)	<0.01 (χ^2^)	22.5 (2; 47)	19.5 (0; 33)	48.5 (31; 53)	<0.05 (#)
10	Other pus	18	13 (72)	5 (28)	ns (χ^2^)	49 (16; 69)	29 (14; 60)	79 (65; 83)	<0.01 (#)
11	Other sources	27	20 (74)	7 (26)	<0.05 (χ^2^)	33 (0; 65)	33 (0; 64)	19 (0; 73)	ns (†)
12	Total	784	395 (50)	389 (50)	na	54.00 (12; 72)	51 (10; 68)	57 (15; 76)	<0.01 (†)

LTR, lower respiratory tracts; FTR, female reproductive tracts; BF, body fluids; POW, postoperative wound; Other pus (pus-derived isolates that could not be assigned to the created groups, e.g., pus from the ear); Other sources (isolates from sources that could not be assigned to the created groups, e.g., from the throat); *n* (%) – number (percent) of *E. coli* isolates from given source; Age – median age of patients; Age_M_ – median age of males; Age_F_ – median age of females; Q1, Q3 – quartiles; *p*-value was measured by *T*-test (#), chi-square (χ^2^) test or Mann–Whitney U test (†); *p* < 0.05 was considered as statistically significant; ns, not statistically significant; na, not applicable.

### 2.2 PCRs

Bacterial cultures were grown for 24 h at 37°C with shaking, at 250–300 rpm in Tryptic Soy Broth (BioMaxima S.A.). The genomic DNA was isolated from 1.5 mL of bacterial culture using GenElute^™^ Bacterial Genomic DNA Kits (Sigma-Aldrich^®^). The concentration of DNA samples was measured with DeNovix (DeNovix Inc.) and diluted with Tris-EDTA (Sigma-Aldrich^®^) solution if necessary. PCRs were performed using approximately 20 ng of bacterial DNA and DreamTaq^™^ Green DNA Polymerase Master Mix (2x) (ThermoFisher Scientific^™^) in Mastercycler^®^ Nexus (Eppendorf, Juelich, Germany).

The VAGs (*papC*, *sfaD/sfaE*, *cnf1*, *usp., fimG/H*, *hlyA*) were identified according to the protocol described previously (Adamus-Białek et al., 2019). Multiplex PCR was performed using 25 μL of aforementioned Master Mix, 10 pmol of each primer (oligo.pl), 1 μL of bacterial DNA and filled with MiliQ water to 50 μL of total volume. The cycling conditions were as follows: initial denaturation at 95°C for 2 min, followed by 35 cycles of 1 min at 95°C, 90 s at 60°C, and 3 min at 72°C, followed by 8 min at 72°C.

The analysis of phylogenetic groups was performed according to the method of Clermont et al. (2013). PCRs mixtures contained 12.5 μL of aforementioned Master Mix, 100 pmol of each primer (oligo.pl), 1 μL of bacterial DNA and filled with MiliQ water to 25 μL of total volume. The cycling conditions for quadruplex-PCR (A, B1, B2, D, F clades) and duplex-PCR (E and C clades) were as follows: initial denaturation at 95° C for 4 min, followed by 30 cycles of 5 s at 95°C, 20 s at temperature annealing (Table 2), and 1 min at 72°C, followed by 5 min at 72°C.

**Table 2:** Amplified regions, oligonucleotide primers, amplicons and PCR conditions, Tan – temperature annealing, bp – base pairs, Ref – a reference to the primer sequence.

Amplified region	Primer name	Primer sequence	PCR (bp)	T_an_ [°C]	References
**Virulence-associated genes**					
*papC*	pap1	GACGGCTGTACTGCAGGGTGTGGCG	328	60	Adamus-Białek et al. (2019)
	pap2	ATATCCTTTCTGCAGGGATGCAATA			
*sfaD/E*	sfa1	CTCCGGAGAACTGGGTGCATCTTAC	410	60	
	sfa2	CGGAGGAGTAATTACAAACCTGGCA			
*cnf1*	cnf1a	AAGATGGAGTTTCCTATGCAGGAG	498	60	
	cnf2a	CATTCAGAGTCCTGCCCTCATTATT			
*usp*	usp1mod	TTCTGGGGAACTGACATTCACGG	657	60	
	usp2mod	CCTCAGGGACATAGGGGGAA			
*fimG/H*	fimGH1	GCAATGTTGGCGTTCGCAAGTGC	1,001	60	
	fimGH2	CGTAAATATTCCACACAAACTGG			
*hlyA*	hly1mod	AACAACGATAAGCACTGTTCTGGCT	1,177	60	
	hly2mod	ACCATATAAGCGGTCATTCCCATCA			
**Phylogenetic groups of *E. coli***					
*chuA*	chuA.1b	ATGGTACCGGACGAACCAAC	288	59	Clermont et al. (2013)
	chuA.2	TGCCGCCAGTACCAAAGACA			
*yjaA*	yjaA.1b	CAAACGTGAAGTGTCAGGAG	211	59	
	yjaA.2b	AATGCGTTCCTCAACCTGT			
*TspE4*.C2	TspE4.C2.1b	CACTATTCGTAAGGTCATCC	152	59	
	TspE4.C2.2b	AGTTTATCGCTGCGGGTCGC			
*arpA*	AceK.f	AACGCTATTCGCCAGCTTGC	400	59	
	ArpA1.r	TCTCCCCATACCGTACGCTA			
*arpA*	ArpAgpE.f	GATTCCATCTTGTCAAAATATGCC	301	57	
	ArpAgpE.r	GAAAAGAAAAAGAATTCCCAAGAG			
*trpA*	trpAgpC.f	AGTTTTATGCCCAGTGCGAG	219	59	
	trpAgpC.r	TCTGCGCCGGTCACGCCC			
*trpA*	trpBA.f	CGGCGATAAAGACATCTTCAC	489	57/59	
	trpBA.r	GCAACGCGGCCTGGCGGAAG			
**CRISPR *E. coli***					
*cas2 iap*	CRISPR1fw	CGTAYYCCGGTRGATTTGGA	–	53	Dang et al. (2013)
	CRISPR1rev	GTTATGCGGATAATGCTACC			
*ygcF ygcE*	CRISPR2fw	GTCGATGCAAACACATAAATA	–	50	
	CRISPR2rev	AAATCGTATGAAGTGATGCAT			
*cas1 clpA*	CRISPR3fw	GCCCACCATTCACCTGT	–	48	
	CRISPR3rev	GCGCTGGATAAAGAGAAAAAT			
*infA cys4*	CRISPR4fw	GTACGACCTGAGCAAAG	–	53	
	CRISPR4rev	CTGAACAGCGGACTGATTTA			

The detection of CRISPR regions (1, 2, 3, 4) was performed according to the protocol described by Dang et al. (2013). Single PCRs mixtures contained 12.5 μL of aforementioned Master Mix, 100 pmol of each primer (oligo.pl), 1 μL of bacterial DNA and filled with MiliQ water to 25 μL of total volume. The cycling conditions of CRISPR 1 and 2 were as follows: initial denaturation at 95°C for 2 min, followed by 30 cycles of 1 min at 95°C, 1 min at annealing temperature (Table 2), and 1 min at 72°C, followed by 5 min at 72°C. The cycling conditions of CRISPR 3 and 4 were as follows: initial denaturation at 95°C for 2 min, followed by 30 cycles of 30 s at 95°C, 30 s at annealing temperature (Table 2), and 30 s at 72°C, followed by 5 min at 72°C.

After electrophoresis on 2% agarose gel, the PCR products were visualized under UV. Primer sequences, amplified regions and annealing temperature are presented in Table 2.

### 2.3 Statistical analyses

Qualitative and quantitative analyzes were carried out using appropriate statistical tests of the Statistica version 13.3 (TIBCO Software Inc.), *p* < 0.05 meant statistically significant. The normality of the distribution was checked with the Shapiro–Wilk test. The Student’s T Test (normal distribution of variables) or the Mann–Whitney U Test (skewed distribution of variables) was used in the following comparisons: the age of patients; the number of VAGs in sputum-derived isolates and other isolates, the CRISPR groups and the number of antibiotics to which isolates were resistant; the CRISPR groups and the number of VAGs. The chi-square test was used in following comparisons: the number of women and men, the studied properties of *E. coli* between urinary-derived isolates and isolates from other clinical groups, urosepsis-derived isolates and blood-derived isolates and urine-derived isolates, the studied properties of *E. coli* and antibiotic resistance; ESBL-producing isolates and MDR isolates in the sputum compared to lower respiratory tract group. The Kruskal-Wallis ANOVA test and *post-hoc* Dunn’s test were used to calculate the following comparisons: the VAGs number in isolates from individual clinical groups; the number of antibiotics to which isolates are resistant from particular clinical groups; the number of antibiotics to which isolates are resistant in specific age ranges of patients (age groups), VAGs in comparison to individual phylogenetic groups; the number of isolates that simultaneously have 1, 2, 3, 4, 5, 6, or 0 VAGs compared to the sum of the number of antibiotics to which individual isolates in given clinical groups are resistant, the isolates that simultaneously have 1, 2, 3, 4, or 0 CRISPR types compared to the sum of the number of VAGs possessed by individual isolates from given clinical groups. GraphPad Prism, version 9 (San Diego, CA, United States) was used for derivation of figures.

## 3 Results

### 3.1 Occurrence of *Escherichia coli* among clinical samples

**Figure 1:**
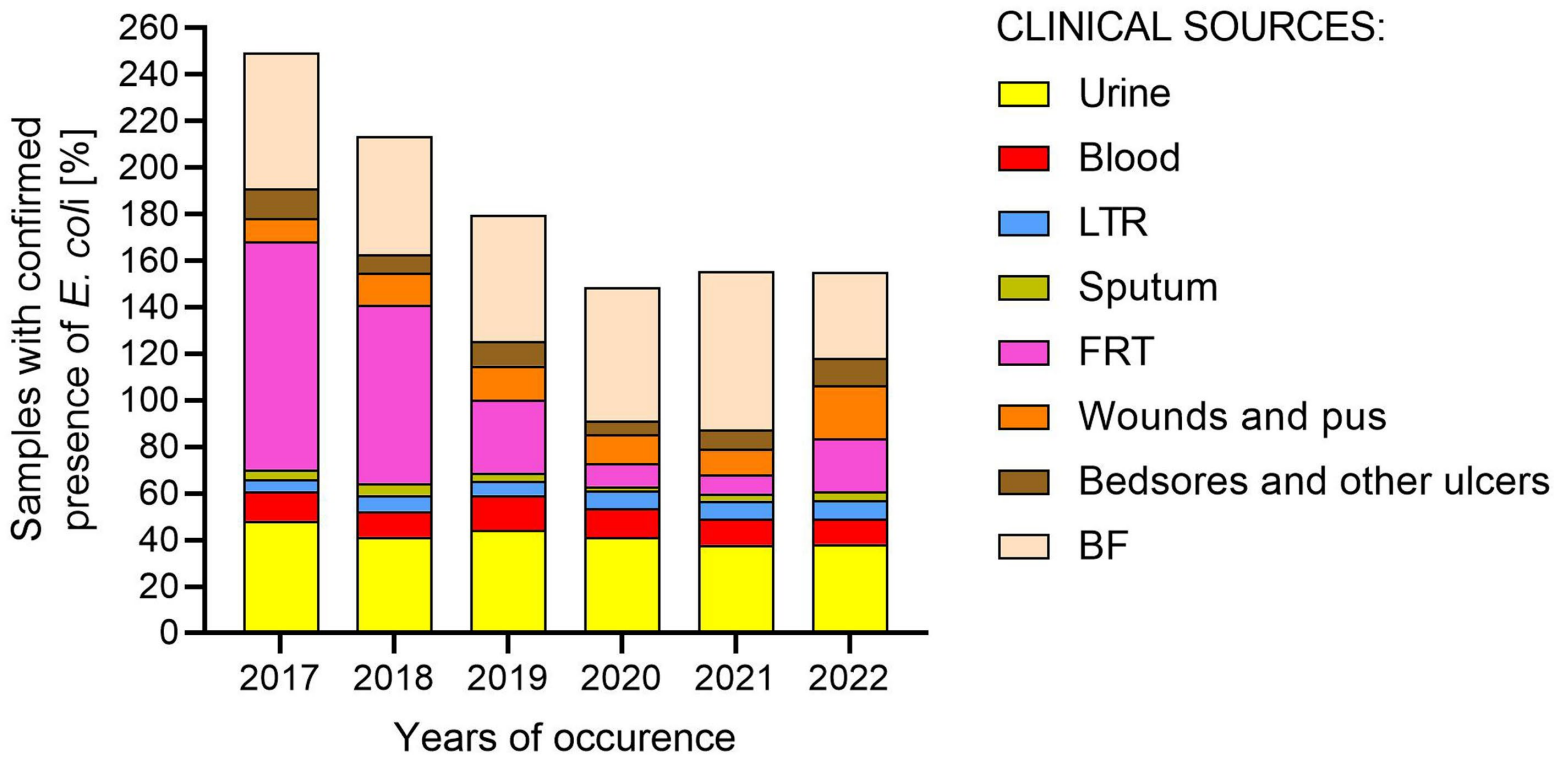
Percentage of samples with confirmed presence of *E. coli* from various clinical materials in 2017–2022. LTR, lower respiratory tracts; FTR, female reproductive tracts; BF, body fluids; POW, postoperative wound.

Firstly, epidemiological data on the frequency of *E. coli* occurrence in clinical materials were collected (Supplementary Table S1). The data concerned the number of all tested samples, the number of samples in which no clinically important/relevant bacteria were detected, and the number of samples with confirmed presence of *E. coli*, collected from patients of Regional Hospital in Kielce in 2017–2022 (Figure 1). The diagnosed samples were assigned to eight clinically relevant groups: urine; blood; lower respiratory tracts (LRT); sputum; bedsores and ulcers; body fluids (BF, group includes infected fluids/pus of bile, abdominal and peritoneal cavity, appendix, abscesses from the abdominal cavity); wounds and pus; and female reproductive tract (FRT). The urine and blood samples were most often sent for microbiological tests. Samples from bedsores and ulcers, wounds and pus, and lower respiratory tracts were most often positive for the presence of clinically important bacteria. *E. coli* was most often isolated in BF and urine (56 and 41% of positive samples, respectively), least often in sputum and lower respiratory tracts (4 and 7% of positive samples, respectively), meaning the prevalence of *E. coli* in the remaining samples ranged from 9 to 13%. In 2017–2021, there has been a decrease in the number of *E. coli* infections in samples from the FRT with a similar number of tested samples and detected general infections (from 98 to 8% of positive samples). However, in 2022 the number of infections increased (23%).

The above epidemiological data show a picture of the spread of infections caused by *E. coli*. For subsequent analysis the studied isolates were based on the specificity of used antibiotics and the clinical picture of the infection divided into the following 11 groups: urine; blood; LRT; sputum; BF; FRT; perianal pus; other pus (pus-derived isolates that could not be assigned to the created groups—e.g., pus from the ear); wound; postoperative wound (POW); and other sources (isolates from sources that could not be assigned to the created groups—e.g., from the throat, ear). Table 1 presents the number of isolates in each clinical group, and the sex and age structure of the patients from whom the materials were collected. Additionally, a group of bacteria causing urosepsis was separated from a blood-derived isolates for some analyses. Overall, the number of male and female samples was very similar (male samples *n* = 395, female samples *n* = 389), however, considering the source of isolation (clinical groups), *E. coli* was more frequently isolated from males in the case of LRT (*p* < 0.001; chi-square test), BF (*p* < 0.05; chi-square test), perianal pus (*p* < 0.05; chi-square test) and other sources (*p* < 0.05; chi-square test). On the contrary, it was more often identified in females in the case of urine (*p* < 0.001; chi-square test).

The median age of all patients was 54 years. In general, the age of females (median = 57) was significantly higher than the age of males (median = 51) (*p* < 0.05; Mann–Whitney U test). In almost all materials (except LTR, POW and other sources), the female group was older than the male group, and significant differences were observed in BF, perianal pus, and other pus and wound. The lowest median age in both females and males was recorded for BF (median = 15), the highest for sputum (median = 80).

### 3.2 Virulence-associated genes profiles

The presence of the *papC, sfaD/E, cnf1, usp., fimG/H* and *hlyA* genes was analyzed among the studied *E. coli* isolates (*n* = 784). VAGs were identified in *E. coli* isolated from all clinical materials, however *E. coli* with highest average virulence score were identified in the urine (Table 3). The number of VAGs was statistically significantly higher in *E. coli* isolated from urine than from: sputum (*p* < 0.05, Kruskal-Wallis test, *post-hoc* Dunn’s test), blood (*p* < 0.05, Kruskal-Wallis test, *post-hoc* Dunn’s test), wounds (*p* < 0.05, Kruskal-Wallis test, *post-hoc* Dunn’s test), BF (*p* < 0.001, Kruskal-Wallis test, *post-hoc* Dunn’s test) and other sources (*p* < 0.05, Kruskal-Wallis test, *post-hoc* Dunn’s test). Another group of isolates with similarly high virulence potential was LRT-derived *E. coli*. The sputum-derived isolates are clearly distinguished; they have the least pathogenic factors in comparison to other *E. coli* isolates (*p* < 0.001; Mann–Whitney U test).

**Table 3:** The average virulence and resistance score in studied *E. coli* isolates.

Clinical materials	Virulence score	Resistance score
Urine	3.4	2.7
Blood	2.7	2.6
LRT	3.3	2.9
Sputum	1.7	7.5
FRT	2.9	1.4
Wound	2.4	2.6
POW	2.4	3.4
BF	2.3	1.5
Perianal pus	3.2	1.5
Other pus	3.1	1.4
Other sources	2.2	2.5

The average virulence/resistance score means the total number of all identified VAGs/antibiotic resistance from isolates in a certain *E. coli* group divided with the number of isolates in the certain *E. coli* group; VAGs, virulence-associated genes; LTR, lower respiratory tracts; FTR, female reproductive tracts; BF, pus of the abdominal cavities; POW, postoperative wound.

**Figure 2:**
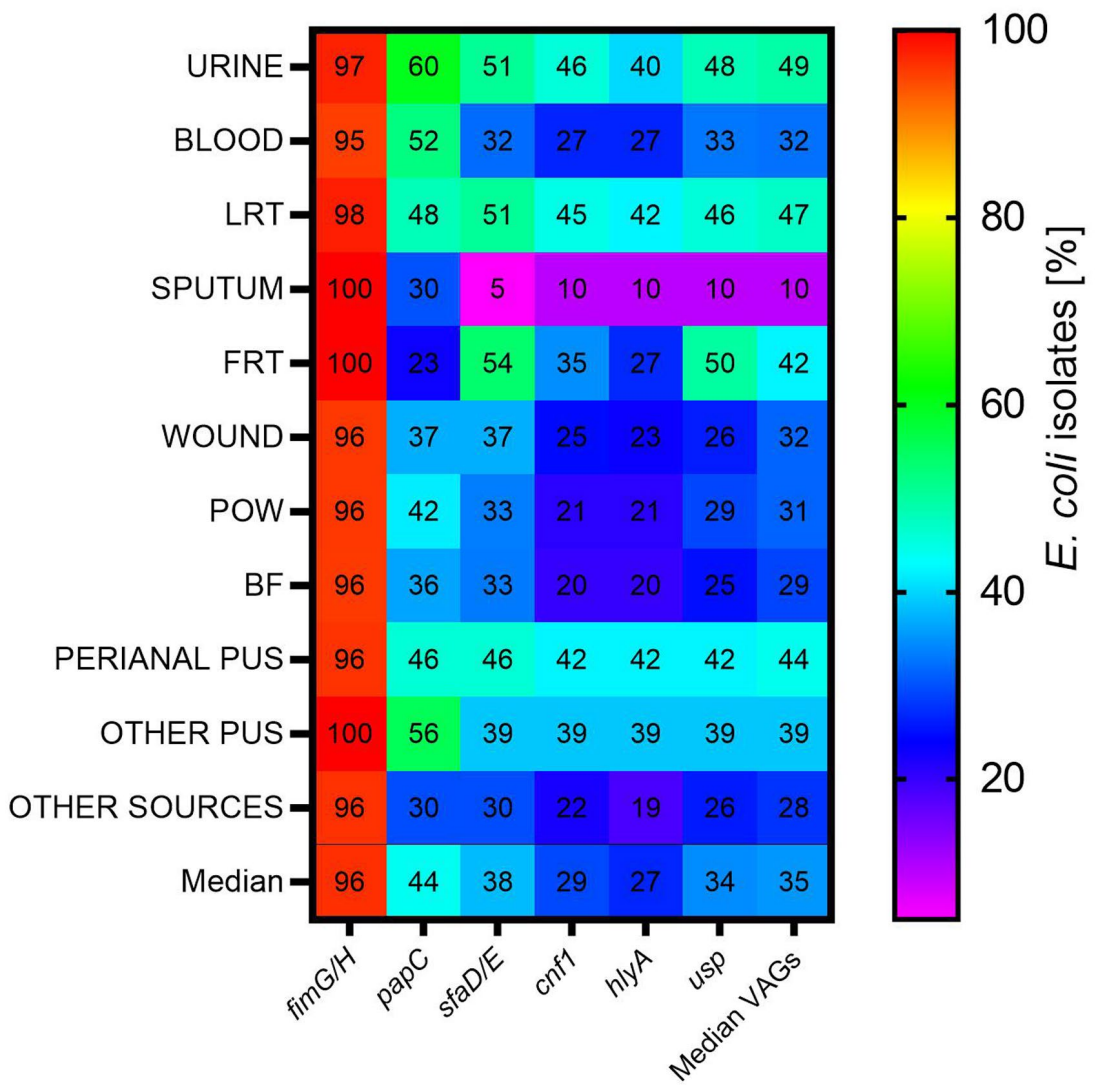
The occurrence of virulence-associated genes among *E. coli* isolates from various clinical materials. LTR, lower respiratory tracts; FTR, female reproductive tracts; BF, body fluids; POW, postoperative wound; Other pus (pus-derived isolates that could not be assigned to the created groups, e.g., pus from the ear); Other sources (isolates from sources that could not be assigned to the created groups, e.g., from the throat).

*fimGH* was the most common among the studied genes—it was present in almost all *E. coli* isolates (median 96.00% of all isolates)—followed by *papC*, *sfaD/E*, *usp., cnf1*, *hlyA* (median 44, 38, 34, 29, and 27%, respectively) (Figure 2). This order was similar for urine, blood, POW and BF. At least one pathogenic factor gene, excluding *fimG/H*, was present in 67% of the tested isolates. *papC* was present most often in urine-derived isolates (60%) and least often in FRT (23%); *sfaD/E* was present most often in FRT (54%) and least often in sputum (5%); *cnf*1 was present most often in urine (46%) and least often in sputum (10%); *hlyA* was present most often in LRT (42%) and least often in sputum (10%); *usp* was present most often in FRT (50%) and least often in sputum (10%).

### 3.3 Phylogenetic groups prevalence

**Figure 3:**
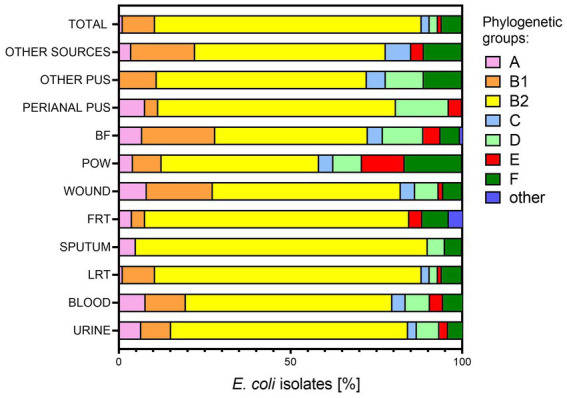
Affiliation to phylogenetic groups of *E. coli* isolates from various clinical materials. LTR, lower respiratory tracts; FTR, female reproductive tracts; BF, body fluids; POW, postoperative wound; Other pus (pus-derived isolates that could not be assigned to the created groups, e.g., pus from the ear); Other sources (isolates from sources that could not be assigned to the created groups, e.g., from the throat); phylogenetic groups A, B1, B2, C, D, E, F.

The entire collection of *E. coli* isolates (*n* = 784) was examined in terms of phylogenetic affiliation. Generally, most of the analyzed isolates belonged to the phylogenetic group B2 (median 65% of all isolates), followed by B1 (median 9%), D (median 6.59%), F (median 6%), A (median 4.58%), C (median 3%), and E (median 3%) (Figure 3). We consider groups I/II and UNKOWN as statistically insignificant, as they were represented by only one representative (*n* = 1; 0.13% each, respectively). A similar division of phylogenetic groups as mentioned above was noted for the urine-derived isolates. Again, sputum group seems to stand out from the rest of the clinical groups—there are only 4 out of 7 typical phylogenetic groups and B2 was represented by 85% of the isolates, the highest among all the clinical groups. The presence of the second most common group B1 was not observed, neither were C and E. The most diverse groups were the POW and BF, where the distribution of the remaining phylogenetic groups in relation to B2 is the largest. Most of the “other” phylogenetic groups were found in FRT-derived isolates, although they still accounted for only 4% of all isolates. Groups C and D were not identified in this group of bacteria. The remaining clinical groups of isolates also differed from each other in the profile of phylogenetic groups.

### 3.4 Antibiotic resistance profiles

**Figure 4:**
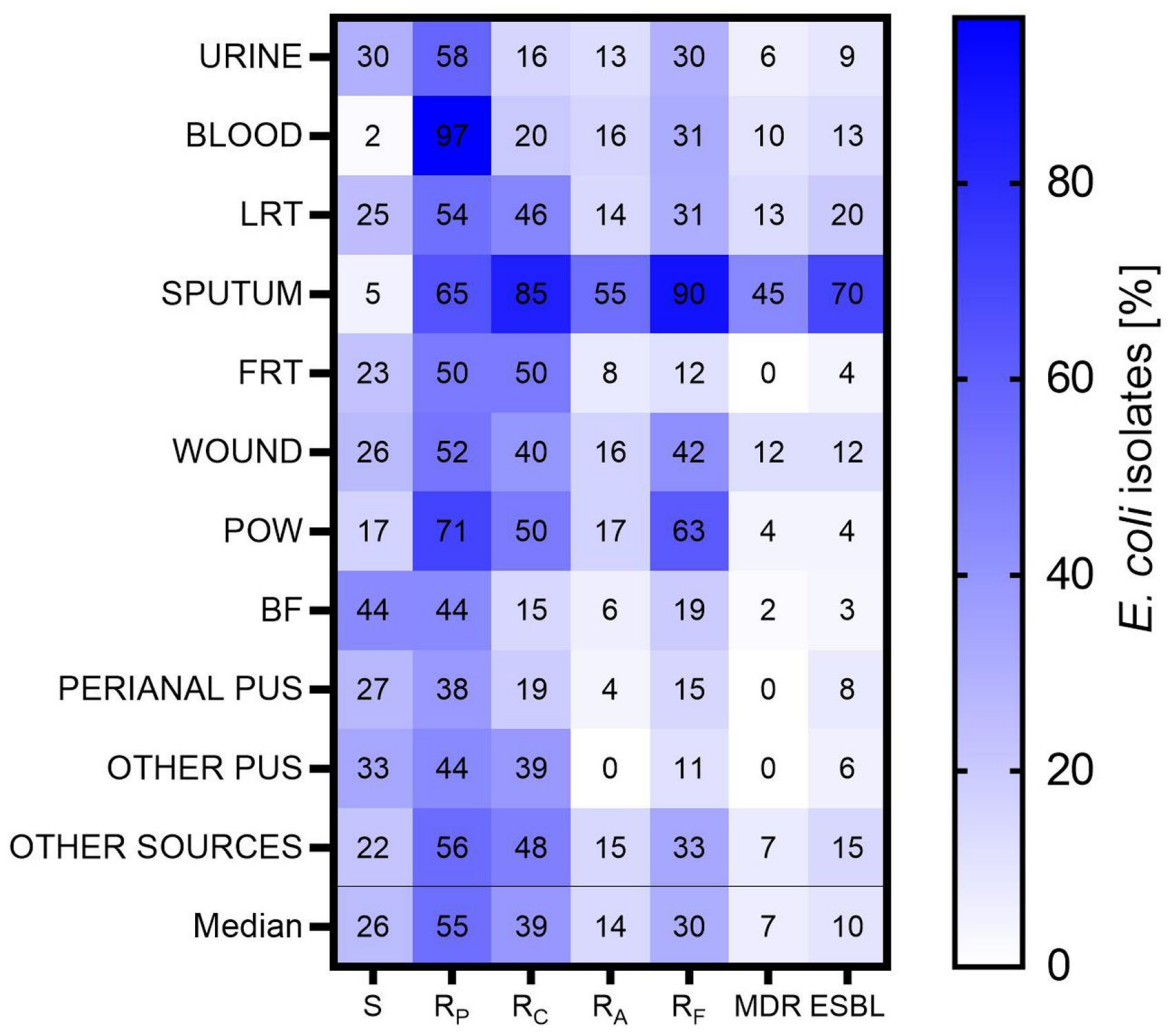
The resistance to particular groups of antibiotics among *E. coli* isolates. LTR, lower respiratory tracts; FTR, female reproductive tracts; BF, body fluids; POW, postoperative wound; Other pus (pus-derived isolates that could not be assigned to the created groups, e.g., pus from the ear); Other sources (isolates from sources that could not be assigned to the created groups, e.g., from the throat); S – isolates sensitive for all antibiotics; R_P_ – isolates resistant to at least one antibiotic from penicillins; R_C_ – isolates resistant to at least one antibiotic from cephalosporins; R_A_ – isolates resistant to at least one aminoglycoside; R_F_ – isolates resistant to at least one fluoroquinolone; MDR – isolates resistant to at least one antibiotic from all three groups (III/IV generations of cephalosporins, aminoglycosides, fluoroquinolones); ESBL – isolates producing ESBL.

Resistance analysis was performed among the groups of antibiotics active against *E. coli*: penicillins (ampicillin, ampicillin/sulbactam, amoxicillin/clavulanate, piperacillin/tazobactam), cephalosporins (cefuroxime, cefotaxime, ceftazidime, cefepime), carbapenems (ertapenem, imipenem, meropenem), monobactams (aztreonam), aminoglycosides (amikacin, gentamicin, tobramycin), fluoroquinolones (norfloxacin, ciprofloxacin, levofloxacin) and antibiotics such as: nitrofurantoin, cotrimoxazole (trimethoprim/sulfamethoxazole), tigecycline, and colistin. Among the analyzed *E. coli* isolates (*n* = 784), the highest sensitivity to all analyzed antibiotics was identified among BF-derived isolates (44% of susceptible BF-derived isolates) and other pus (33%), the least sensitive to all antibiotics was identified among blood and sputum-derived isolates (2 and 5%, respectively) (Figure 4). Urine-derived isolates were resistant to significantly more antibiotics than BF-derived isolates (*p* < 0.05; Kruskal-Wallis test, *post-hoc* Dunn’s test), and no similar correlation with the other groups was observed. Interestingly, sputum-derived isolates were the most resistant group in comparison to all clinical materials (in the range from *p* < 0.05 to *p* < 0.001; Kruskal-Wallis test, *post-hoc* Dunn’s test), except POW.

The studied *E. coli* isolates showed the greatest sensitivity to aminoglycosides (Figure 4). It is worth noting 55% of all isolates and almost all blood-derived isolates were resistant to at least one antibiotic of penicillin. Also, a high percentage of penicillin resistance was found in the group of POW (71%), the lowest—among perianal pus (35%). In the case of cephalosporins—the highest resistance was observed for sputum-derived isolates (85%) and the lowest for BF-derived isolates (15%). In the group of aminoglycosides—the highest resistance was observed again for sputum-derived isolates (75%), the lowest for perianal pus-derived isolates (4%). Any other pus-derived isolates were resistant to aminoglycosides. Similarly, in the group of fluoroquinolones—the highest for sputum (90%), the lowest for other pus (11%).

All tested *E. coli* isolates were sensitive to carbapenem antibiotics (data not shown). Most often, MDR was expressed by sputum-derived isolates (45%); no MDR isolates were found among isolates from the FRT, perianal pus and other pus. The ESBL production was most often expressed by isolates from sputum (70%), least often from BF (3%). The results for two physiologically similar groups—sputum and lower respiratory tract—were compared. It was observed that sputum-derived isolates were 9.33 times more likely (Odds Ratio; *p* < 0.001, chi-square test) than LRT-derived isolates to express the ESBL, and 5.51 times more likely to be MDR-type isolates (Odds Ratio, *p* < 0.001, chi-square test).

There is a statistically significant difference between the drug resistance of *E. coli* and the age of the patient. *E. coli* isolated from younger patients (<25 years old) were statistically significantly more sensitive (resistant to fewer antibiotics) than isolates from older patients (50–74 years old) and seniors (>74 years old) (*p* < 0.001, Kruskal-Wallis Test, *post-hoc* Dunn’s test). The number of antibiotics to which a given isolate was resistant did not correlate with the sex of the patient.

### 3.5 CRISPR-*Cas* regions occurrence

**Figure 5:**
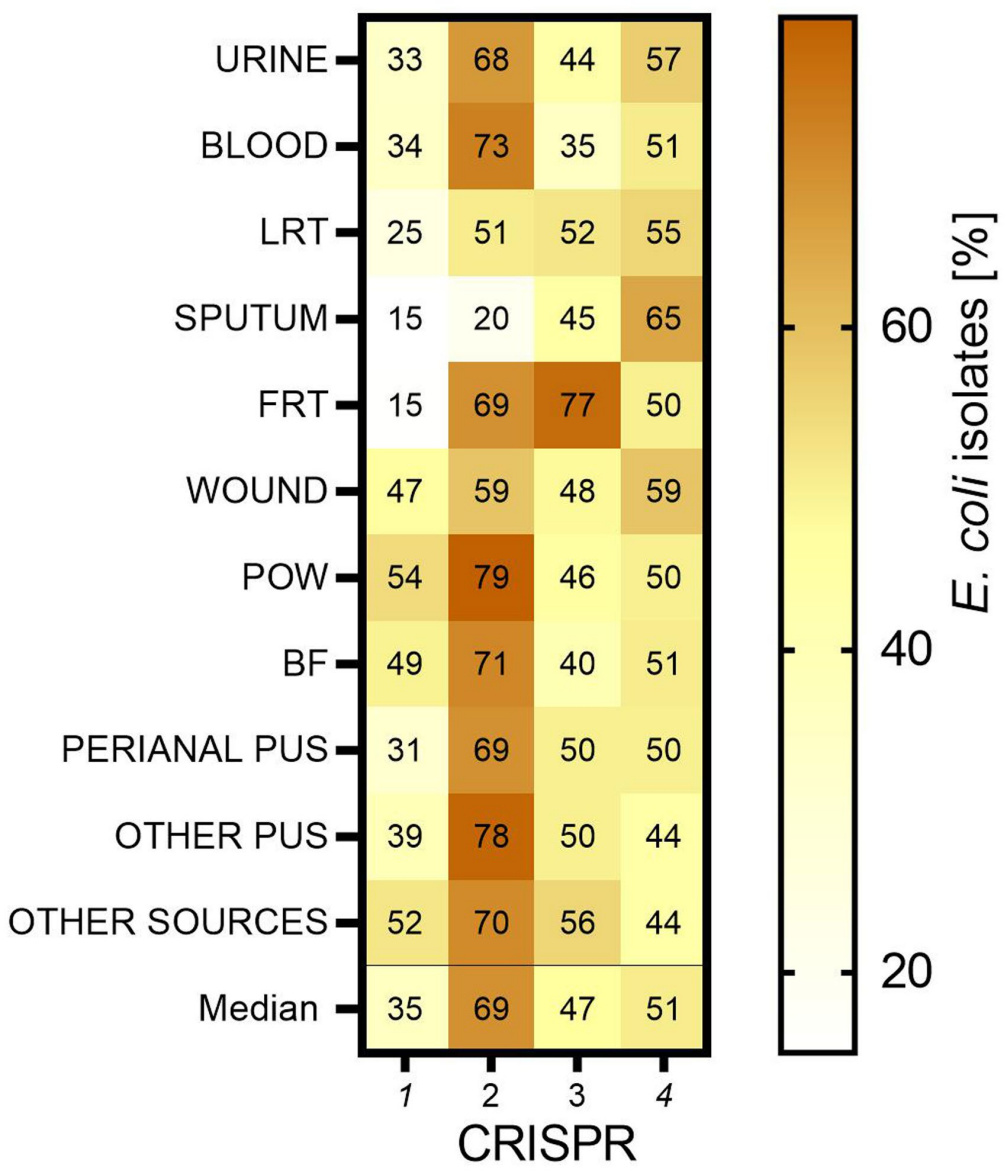
The occurrence of CRISPR-*Cas* regions in *E. coli* isolates. LTR, lower respiratory tracts; FTR, female reproductive tracts; BF, body fluids; POW, postoperative wound; Other pus (pus-derived isolates that could not be assigned to the created groups, e.g., pus from the ear); Other sources (isolates from sources that could not be assigned to the created groups, e.g., from the throat).

Four different CRISPR-*Cas* type regions were analyzed for all collected *E. coli* isolates (*n* = 784). The greatest number of *E. coli* isolates possessed CRISPR 2 (median 69% of all isolates)—this predominance was statistically significant (*p* < 0.001; Mann–Whitney U test)—followed by CRISPR 4 (median 51%), CRISPR 3 (median 47%) and the smallest prevalence was detected for CRISPR 1 (median 35%) (Figure 5). Regarding individual clinical groups, *E. coli* isolates with CRISPR 1 were most common in the group of POW (54%), the least in FRT (15%). Again, isolates with CRISPR 2 were most common in POW (79%), the least in sputum (20%). Those with CRISPR 3 were most common in FRT (77%), the least in blood (35%). CRISPR 4 was the most commonly present in the sputum-derived isolates (65%), the least in the other group (44%).

### 3.6 Comparison of urine-derived *Escherichia coli* to other isolates

We compared the *E. coli* urine-derived isolates to *E. coli* isolated from other clinical materials in order to find significant differences that could characterize uropathogens (Table 4; Supplementary Table S2). It was shown that significantly urine-derived isolates were more resistant and virulent than BF-derived isolates, and they were more sensitive and virulent than sputum-derived isolates. It is also clear visible that urine-derived isolates were more often sensitive to cephalosporins than other *E. coli* isolates. Generally, urine-derived isolates were also significantly more virulent than other isolates. No correlation was observed for *fimG/H*. In the case of the phylogenetic affiliation, the B2 group was significantly more frequent among urine-derived isolates than in wound, POW and BF. Conversely, the B1 group was significantly more frequent in wound and BF, then, E and F—they were found more often in POW. CRISPR 1 region was found significantly more often in wound, POW and BF-derived isolates than in urine-derived isolates, whereas CRISPR 2 region was present more often in urine-derived isolates than LRT and sputum-derived isolates. No correlation has been reported for CRISPR 4.

**Table 4:** The correlation between urine-derived *E. coli* isolates and *E. coli* isolated from other clinical materials in relation to a given features.

	Clinical materials																						
Feature	Urine (%)	Blood (%)	χ^2^	LRT (%)	χ^2^	Sputum (%)	χ^2^	FRT (%)	χ^2^	Wound (%)	χ^2^	POW (%)	χ^2^	BF (%)	χ^2^	Perianal pus (%)	χ^2^	Other pus (%)	χ^2^	Other sources (%)	χ^2^	All (%)	χ^2^
*papC*	59.9	52.3	ns	48.2	ns	30.0	*	23.1	*	37.0	ns	41.7	ns	36.3	*	46.2	ns	55.6	ns	29.6	*	41.7	*
*sfaD/E*	50.8	32.0	*	50.6	ns	5.0	*	53.9	ns	37.0	ns	33.3	ns	33.1	*	46.2	ns	38.9	ns	29.6	*	36.5	*
*cnf1*	45.7	26.6	*	44.7	ns	10.0	*	34.6	ns	24.7	ns	20.8	*	20.0	*	42.3	ns	38.9	ns	22.2	*	27.6	*
*usp*	48.2	32.8	*	45.9	ns	10.0	*	50.0	ns	26.1	ns	29.2	ns	25.0	*	42.3	ns	38.9	ns	26.0	*	31.9	*
*hlyA*	40.1	26.6	*	42.4	ns	10.0	*	27.0	ns	23.3	ns	20.8	ns	20.0	*	42.3	ns	38.9	ns	18.6	*	26.6	*
*fimG/H*	97.5	95.3	ns	97.7	ns	100.0	ns	100.0	ns	95.9	ns	95.8	ns	95.6	ns	96.2	ns	100.0	ns	96.3	ns	96.4	ns
A	6.6	7.8	ns	1.2	ns	5.0	ns	3.9	ns	8.2	ns	4.2	ns	6.9	ns	7.7	ns	0.0	ns	3.7	ns	5.8	ns
B1	8.6	11.7	ns	9.4	ns	0.0	ns	3.9	ns	19.2	(*)	8.3	ns	21.3	(*)	3.9	ns	11.1	ns	18.5	ns	14.0	ns
B2	69.0	60.2	ns	77.7	ns	85.0	ns	77.0	ns	54.8	*	45.8	*	44.4	*	69.2	ns	61.1	ns	55.6	ns	59.0	ns
C	2.5	3.9	ns	2.4	ns	0.0	ns	0.0	ns	4.1	ns	4.2	ns	4.4	ns	0.0	ns	5.6	ns	7.4	ns	3.6	ns
D	6.6	7.0	ns	2.4	ns	5.0	ns	0.0	ns	6.9	ns	8.3	ns	11.9	ns	15.4	ns	11.1	ns	0.0	ns	7.5	ns
E	2.5	3.9	ns	1.2	ns	0.0	ns	3.9	ns	1.4	ns	12.5	(*)	5.0	ns	3.9	ns	0.0	ns	3.7	ns	3.6	ns
F	4.1	5.5	ns	5.9	ns	5.0	ns	7.7	ns	5.5	ns	8.2	(*)	5.6	ns	0.0	ns	11.1	ns	11.1	ns	6.3	ns
S	30.0	1.6	*	24.7	ns	5.0	*	23.1	ns	26.0	ns	16.7	ns	43.8	(*)	27.0	ns	33.3	ns	22.2	ns	24.2	ns
R_P_	57.9	96.9	(*)	54.1	ns	65.0	ns	50.0	ns	52.1	ns	70.8	ns	44.4	*	38.5	ns	44.4	ns	55.6	ns	60.5	ns
R_C_	15.7	20.3	ns	45.9	(*)	85.0	(*)	50.0	(*)	39.7	(*)	50.0	(*)	15.0	ns	19.2	ns	38.9	(*)	48.2	(*)	31.5	(*)
R_A_	12.7	15.6	ns	14.1	ns	55.0	(*)	7.7	ns	16.4	ns	16.7	ns	6.3	*	3.9	ns	0.0	ns	14.8	ns	12.9	ns
R_F_	30.0	31.3	ns	30.6	ns	90.0	(*)	11.5	*	42.5	ns	62.5	(*)	19.4	*	15.4	ns	11.1	ns	33.3	ns	30.5	ns
ESBL	9.1	13.3	ns	20.0	ns	70.0	(*)	3.9	ns	12.3	ns	4.2	ns	3.1	*	7.7	ns	5.6	ns	14.8	ns	12.1	ns
MDR	6.1	10.2	ns	13.0	ns	45.0	(*)	0.0	ns	12.3	ns	4.2	ns	1.9	*	0.0	ns	0.0	ns	7.4	ns	8.2	ns
C1	33.0	33.6	ns	24.7	ns	15.0	ns	15.4	ns	46.6	(*)	54.2	(*)	49.4	(*)	30.8	ns	38.9	ns	51.9	ns	38.5	ns
C2	67.6	72.7	ns	50.6	*	20.0	*	69.2	ns	58.9	ns	79.2	ns	71.3	ns	69.2	ns	77.8	ns	70.4	ns	65.6	ns
C3	43.7	35.2	ns	51.8	ns	45.0	ns	76.9	(*)	48.0	ns	45.8	ns	40.0	ns	50.0	ns	50.0	ns	55.6	ns	45.1	ns
C4	56.9	50.8	ns	55.3	ns	65.0	ns	50.0	ns	58.9	ns	50.0	ns	50.6	ns	50.0	ns	44.4	ns	44.4	ns	52.3	ns

LTR – lower respiratory tracts; FTR – female reproductive tracts; BF – pus of the abdominal cavities; POW – postoperative wound; All – all other than urine materials; S – isolates sensitive for all antibiotics; R_P_ – isolates resistant to at least one antibiotic from penicillins; R_C_ – isolates resistant to at least one antibiotic from cephalosporins; R_A_ – isolates resistant to at least one aminoglycoside; R_F_ – isolates resistant to at least one fluoroquinolone; MDR – isolates resistant to at least one antibiotic from every of three groups (III/IV generations of cephalosporins, aminoglycosides, fluoroquinolones); ESBL – isolates producing ESBL; *papC, sfaD/E, cnf1, usp., hlyA, fimG/H* – virulence-associated genes; A, B1, B2, C, D, E, F – phylogenetic groups; C1, C2, C3, C4 – CRISPR1, CRISPR2, CRISPR3, CRISPR4; χ^2^ – chi-square test used for statistical significance analysis, *p* < 0.05 was considered as statistically significant; * – feature was significantly more often found in urine-derived isolates; (*) – feature was significantly less often found in urine-derived isolates; ns – not statistically significant.

### 3.7 Urosepsis-causing isolates

Among the blood-derived *E. coli,* we identified 28 isolates that caused urosepsis and they were analyzed individually (Table 5; Supplementary Table S3). It should be noted that most of the isolates were resistant to at least one antibiotic from the penicillin group (90%); the group of isolates was characterized by low resistance to other antibiotics (on average 16%) and 100% of the isolates were sensitive to carbapenem antibiotics. It is worth adding that 7% of the isolates were MDR and 11% were ESBL-positive. In the case of VAGs—71.43% of them possessed at least one VAG gene besides *fimG/H*, the most commonly present was *papC.* The vast majority of these isolates belonged to phylogenetic group B2 (75%), next B1 and F were present in 10% of these isolates. The prevalence of CRISPR region was similar to the majority of isolates (the most commonly present was CRISPR 2). The urosepsis-derived isolates were compared to urine-derived and blood-derived isolates (Table 5; Supplementary Table S3). We identified statistically significantly fewer urosepsis-positive isolates resistant to penicillins than blood-derived, but more than urine-derived. Additionally, fewer urosepsis-positive isolates possessed *cnf1* in comparison to urine.

**Table 5:** The properties of urosepsis-positive *E. coli* isolates in correlation to blood-derived and urine-derived *E. coli* isolates.

	Clinical materials				
Feature	Urosepsis (%)	Blood (%)	χ^2^	Urine (%)	χ^2^
*papC*	60.7	52.3	ns	59.9	ns
*sfaD/E*	32.1	32.0	ns	50.8	ns
*cnf1*	25.0	26.6	ns	45.7	*
*usp*	35.7	32.8	ns	48.2	ns
*hlyA*	25.0	26.6	ns	40.1	ns
*fimG/H*	100.0	95.3	ns	97.5	ns
A	0.0	7.8	ns	6.6	ns
B1	10.7	11.7	ns	8.6	ns
B2	75.0	60.2	ns	69.0	ns
C	3.6	3.9	ns	2.5	ns
D	3.6	7.0	ns	6.6	ns
E	7.1	3.9	ns	2.5	ns
F	10.7	5.5	ns	4.1	ns
S	3.6	1.6	ns	30.0	*
R_P_	89.3	96.9	*	57.9	(*)
R_C_	17.9	20.3	ns	15.7	ns
R_A_	10.7	15.6	ns	12.7	ns
R_F_	21.4	31.3	ns	30.0	ns
ESBL	10.7	13.3	ns	9.1	ns
MDR	7.1	10.2	ns	6.1	ns
C1	28.6	33.6	ns	33.0	ns
C2	82.1	72.7	ns	67.6	ns
C3	50.0	35.2	ns	43.7	ns
C4	53.6	50.8	ns	56.9	ns

S – sensitive isolates for all antibiotics; R_P_ – resistant isolates to at least one antibiotic from the penicillins group; R_C_ – resistant isolates to at least one antibiotic from the cephalosporins group; R_A_ – resistant isolates to at least one aminoglycoside; R_F_ – resistant isolates to at least one fluoroquinolone; MDR – resistance to at least one antibiotic from all three groups (III/IV generations of cephalosporins, aminoglycosides, fluoroquinolones); ESBL – isolates producing ESBL; *papC, sfaD/E, cnf1, usp., hlyA, fimG/H* – virulence-associated genes; A, B1, B2, C, D, E, F – phylogenetic groups; C1, C2, C3, C4 – CRISPR1, CRISPR2, CRISPR3, CRISPR4; χ^2^ – chi-square test used for statistical significance analysis, *p* < 0.05 was considered as statistically significant; * – feature was significantly more often found in urosepsis-derived isolates; (*) – feature was significantly less often found in urosepsis-derived isolates; ns – not statistically significant.

### 3.8 Correlations of the studied bacterial features among *Escherichia coli* isolates from different clinical groups

We also investigated the associations between the individual features of the studied *E. coli* isolates. A clear correlation between the resistance and virulence potential was observed. The number of antibiotics to which individual bacterial isolate was resistant was compared to the number of identified VAGs. Isolates with 1 or 2 VAGs are more likely to be resistant to a higher number of antibiotics than isolates with 6 VAGs (*p* < 0.05, respectively; Kruskal-Wallis Test, *post-hoc* Dunn’s test). Generally, the increasing resistance was correlated with the decreasing virulence potential. The presence of a given VAGs among the tested isolates correlated negatively with the number of antibiotics to which the isolates were resistant: *papC* (*p* < 0.05; Mann–Whitney U test), *sfaD/E* (*p* < 0.001; Mann–Whitney U test)*, cnf1* (*p* < 0.001; Mann–Whitney U test), *usp* (*p* < 0.001; Mann–Whitney U test), and *hlyA* (*p* < 0.001; Mann–Whitney U test). In addition, the higher resistance was correlated with the reduced frequency of CRISPR 2 (*p* < 0.001; Mann–Whitney U test), and the increased frequency of CRISPR 4 (*p* < 0.001; Mann–Whitney U test). Most MDR and ESBL-positive isolates belonged to phylogenetic group B2 (87 and 90%, respectively).

It should be noted that CRISPR 1 was the least common in phylogenetic group B2 (5.4%), and the most common in C (100%) and B1 (98%). CRISPR 2 was the least common in the B2 group (50.6%) and the most common in D, A, B1, and E (> 90%). CRISPR 3 was least common in the C group (15.38%), and the most common in A and B2 (>50%). CRISPR 4 was least common in the F group (28.89%), and the most common in D (81%). We recorded isolates that simultaneously had all four CRISPRs (7.3%), belonging to different phylogenetic groups. On the other hand, we identified isolates without the any CRISPR regions (10.5%), with almost all of them belonging to the B2 group (97.6%). We also observed that in the case of group B2, the vast majority of isolates with CRISPR 4 did not have CRISPR 1 (91.5%).

A negative correlation between the number of VAGs and presence of CRISPR 1 and CRISPR 4 was observed (*p* < 0.001, Mann–Whitney U test). No such relation was found for CRISPR 2 and CRISPR 3. We observed that isolates with a lower number of CRISPR regions have significantly more VAGs than isolates with a higher number of CRISPR regions (*p* < 0.001, Kruskal-Wallis Test, *post-hoc* Dunn’s test). The isolates belonging to phylogenetic group B2 have significantly more VAGs than the isolates belonging to the other phylogenetic groups (*p* < 0.001 for each comparison, Kruskal-Wallis Test, *post-hoc* Dunn’s test).

Isolates were also compared between the resistance to particular groups of antibiotics and other features (Table 6; Supplementary Table S4), and these observations were in line with the described above. Generally, lower resistance was correlated with higher virulence potential, especially in the case of fluoroquinolones, excluding *fimG/H*. Regarding the correlation between phylogenetic groups and resistance, the study authors observed the correlation only in the case of the groups B1 and B2. Isolates resistant to fluoroquinolones belonged more often to group B1, but those which were sensitive belonged more often to B2. Conversely, the group B2 of isolates were more resistant to aminoglycosides, but B1 were more sensitive to aminoglycosides and cephalosporins. In the case of other antibiotics (cephalosporins, aminoglycosides, fluoroquinolones), a clear positive correlation was observed for CRISPR 2 and a negative correlation for CRISPR 4. CRISPR 1 and CRISPR 3 also revealed a correlation with the individual antibiotic groups. Penicillins did not show a correlation with phylogenetic affiliation and CRISPR regions of the studied isolates.

**Table 6:** The correlation between the resistance to the antibiotic groups and other features of *E. coli* isolates.

	Penicillins			Cephalosporins			Aminoglycosides			Fluoroquinolones		
Feature	R (%)	S (%)	χ^2^	R (%)	S (%)	χ^2^	R (%)	S (%)	χ^2^	R (%)	S (%)	χ^2^
*papC*	43.9	49.8	ns	44.4	47.0	ns	45.5	46.4	ns	29.8	53.5	(*)
*sfaD/E*	34.1	48.9	(*)	25.0	45.8	(*)	16.8	43.5	(*)	15.1	50.9	(*)
*cnf1*	29.2	36.5	(*)	28.2	33.6	ns	31.7	32.2	ns	13.5	40.3	(*)
*usp*	32.6	41.0	(*)	22.7	41.0	(*)	17.8	38.7	(*)	8.8	47.8	(*)
*fimG/H*	96.4	97.1	ns	96.8	96.7	ns	99.0	96.3	ns	96.2	96.9	ns
*hlyA*	26.0	35.9	(*)	23.6	32.4	(*)	22.8	31.0	ns	8.0	39.6	(*)
A	6.0	6.0	ns	6.5	5.8	ns	3.0	6.4	ns	8.4	5.0	ns
B1	13.9	10.8	ns	8.8	14.1	(*)	4.0	13.9	(*)	20.2	9.3	*
B2	59.7	64.1	ns	66.2	59.7	ns	74.3	59.6	*	49.2	66.9	(*)
C	4.1	2.2	ns	1.9	3.9	ns	3.0	3.4	ns	4.2	2.9	ns
D	7.5	7.0	ns	7.4	7.2	ns	6.9	7.3	ns	5.9	7.9	ns
E	3.4	3.2	ns	3.3	3.4	ns	3.0	3.4	ns	3.8	3.1	ns
F	5.3	6.4	ns	6.0	5.6	ns	5.9	5.7	ns	8.4	4.6	*
C1	38.0	35.9	ns	32.4	38.9	ns	25.7	38.8	(*)	50.8	31.1	*
C2	67.2	64.4	ns	46.8	73.4	(*)	32.7	71.0	(*)	55.0	70.9	(*)
C3	42.2	48.6	ns	54.2	41.2	*	40.6	45.4	ns	39.5	47.1	(*)
C4	55.0	51.0	ns	62.0	50.2	*	65.4	51.7	*	61.8	49.8	*

*papC, sfaD/E, cnf1, usp., fimG/H, hlyA* – virulence-associated genes; A, B1, B2, C, D, E, F – phylogenetic groups; C1, C2, C3, C4 – CRISPR1, CRISPR2, CRISPR3, CRISPR4; R – percentage of resistant *E. coli* isolates; S – percentage of sensitive *E. coli* isolates; χ^2^ – chi-square test used for statistical significance analysis, *p* < 0.05 was considered as statistically significant; * – feature was significantly more often found in resistant isolates; (*) – feature was significantly less often found in resistant isolates; ns – not statistically significant.

## 4 Discussion

### 4.1 Epidemiology of clinical *Escherichia coli* isolates

The cosmopolitan species of *Escherichia coli* is known for its remarkable adaptability to the environment. It is possible thanks to, for example, horizontal gene transfer, modification of the cell wall, regulation of gene expression, molecular mimicry, or various mechanisms of drug resistance (Nielsen et al., 2016; Phillips et al., 2016; Borkowski et al., 2018; Westphal et al., 2018; Vasilakou et al., 2020). Environmental pressure and own fitness have led to the evolution of many *E. coli* strains often causing life-threatening infections. In order to facilitate the monitoring of them, a nomenclature of various intestinal (IPEC) and extraintestinal (ExPEC) pathotypes of *E. coli* has been named (Jesser and Levy, 2020; Riley, 2020; Geurtsen et al., 2022). In our study, we focused on the spread of ExPEC among inpatients, and what is their virulence potential profile, especially compared to UPEC. We can agree with other authors that there is no clinical material in which *E. coli* has never appeared (Allocati et al., 2013; Ruiz-Hernández et al., 2020; Begier et al., 2021; Rathbun et al., 2022). It turned out that *E. coli* was the most prevalent for body fluids infections and urine, however in the case of wounds, ulcers, lower respiratory tract, and postoperative sites, it may be related with nosocomial infections (Figure 1). Studies by other authors confirm that the most common *E. coli* infections are idiopathic bacterial peritonitis and UTI (Cereto et al., 2003; Facciorusso et al., 2019). It should also be noted that the marked decrease in FRT infections caused by *E. coli* (from almost 100 to 8%) was related to the internal policy of the hospital, which began to conduct an antibiogram only for pregnant patients (information confirmed by Department of Microbiology, Regional Hospital, Kielce, Poland).

The studied cohort revealed that in some cases of infection there is significant gender predominance (Table 1). A female predominance in urinary tract infections is often recorded (Guglietta, 2017; Lee et al., 2018), however a male predominance in other infections is puzzling. The more frequent diagnosis of *E. coli* in men with LTR infections may be related to differences in anatomical structure, hormonal balance, and smoking (Marrie et al., 2003; O’Meara et al., 2005; Vrbova et al., 2005; Thomsen et al., 2006; Falagas et al., 2007; Kyu et al., 2022). Probably these factors can be important in the case of other male-dominated infections. What is more, we observed that females were more likely to be older than males for *E. coli* infections, especially for wound and pus. This may be related to the fact that women use medical first aid more often than men (Schlichthorst et al., 2016), therefore their infections can be treated on an outpatient basis at an early stage.

### 4.2 Virulence potential and phylogenetic origin of clinical *Escherichia coli* isolates

Bacterial pathogens are defined by the presence of virulence factors and the ability to cause infection. Uropathogenicity of *E. coli* isolates is also described by the presence of specific virulence-associated genes more often than in other pathotypes. Scientists have proven that UPEC strains are characterized by exceptional genome plasticity and the ability to acquire features that allow them to survive in an unfavorable environment (Naves et al., 2008; Sarowska et al., 2019; Klein and Hultgren, 2020). Many authors point to the presence of UPEC-specific (“urospecific”) virulence factors (Sarowska et al., 2019; Yazdanpour et al., 2020; Bunduki et al., 2021). The most frequently mentioned are fimbriae (mainly P and S), toxins (e.g., cytotoxic necrosis factor type 1, hemolysin A) and less often—bacteriocin *usp* (uropathogenic specific protein); they help them survive in the unfavorable conditions of the urinary tract. Indeed, we identified the genes encoding these factors significantly more often in urinary-derived *E. coli* isolates (Table 4; Supplementary Table S2), but they were also detected in all clinical groups of *E. coli* (Table 3; Figure 2), which is consistent with other authors (Krawczyk et al., 2015; Kim and Lee, 2022; Onlen Guneri et al., 2022), which demonstrates the remarkable plasticity of the UPEC genes. Of particular interest is the high virulence potential profile similarity regarding the LRT-group originating from an anatomically distant site in relation to the isolates from urine. Since *E. coli* is a relatively rare respiratory pathogen, it seems that this topic of research has not been exhausted. Therefore, it seems that the prevalence of VAGs is quite flexible and does not matter in differentiating UPEC from other ExPECs, rather the induction of their expression by the host (Hua et al., 2004; Ireland et al., 2020; Vlková and Silander, 2022). There are known studies showing specific conditions of hosts favoring *E. coli* infections (Sumati and Saritha, 2009; Hannan et al., 2012; O’Brien et al., 2016). In view of the above results, it seems reasonable to ask whether the host environment is responsible for the expression of individual VAG in *E. coli*, and thus the occurrence of UTI (Chen et al., 2013; Bielecki et al., 2014; Sintsova et al., 2019; Frick-Cheng et al., 2020). The presence of pathogenic factors does not always have to be associated with a more severe course of the disease, but their presence certainly increases such a risk. Monitoring of *E. coli* with a high pathogenic potential is justified.

Additionally, we confirmed the previously observed negative association between virulence potential and resistance (Harwalkar et al., 2014; Adamus-Białek et al., 2019), moreover regardless of origin. In the case of phylogenetic affiliation, group B2 was specific among virulent isolates, which may indicate high plasticity of these bacteria genome (Piatti et al., 2008; Wang et al., 2013; Schreiber et al., 2017). Moreover, it was also the most common phylogenetic group among all studied isolates. It is known that UPEC strains are characterized by geographic phylogenetic diversity. In Europe and the United States, group B2 dominates among UPEC, which was confirmed by our research, whereas in Asia most of these strains belong to group D (Piatti et al., 2008; Ejrnæs et al., 2011; Wang et al., 2014). We can thus ask the question whether the phylogenetic group B2 of *E. coli* is the one that has the best adaptive capabilities and thus the pathogenic potential?

Urosepsis-derived isolates seem to be a particularly clinically important group of *E. coli*. However, we did not find features that would clearly distinguish these isolates (Table 5; Supplementary Table S3), which is in line with other authors (Mcnally et al., 2013; Kim and Lee, 2022). Increased resistance to penicillins in this group may only result from their more frequent use, and here we want to draw attention to BF-derived isolates. They are distinctly different from the urine isolates in terms of both lower virulence potential and lower antibiotic resistance, and more frequent occurrence of B1 (Table 4; Supplementary Table S2). In view of the above, we can assume that these are largely isolates originally inhabiting the intestine, being commensals which, as a result of reduced immunity and/or injuries, caused abdominal cavity infection. Many authors have observed the phenomenon of loss of VAGs resulting in the acquisition of drug resistance (Adamus-Białek et al., 2019; Cepas and Soto, 2020). The effect of this phenomenon was probably observed retrospectively in the case of many groups of clinical isolates analyzed in our studies, such as blood-derived, sputum-derived and isolates from other sources.

### 4.3 Drug resistance among clinical *Escherichia coli* isolates

Considering the treatment process and its success, it is difficult to say what is more important—virulence potential or sensitivity to antibiotics? Our observations show that both elements are equally important and interdependent. Overall, we recorded drug resistance at an average level of 35%, but resistance to penicillins is alarming, especially in the group of blood-derived isolates (almost 100%) (Figure 4). Although penicillins are no longer routinely used to treat bacteremia (Mandell et al., 2007; Solomkin et al., 2010; Vallés et al., 2011), they are still commonly used to treat other infections (Vazouras et al., 2020). Therefore, it is very important to constantly monitor the epidemiological situation to react as quickly as possible, among others by limiting the local use of penicillins in empirical therapy, as well as other antibiotics. We also noted a high resistance to cephalosporins which, especially in the context of Gram-negative ESBL-producing bacilli, represent a very serious medical problem. In ESBL-positive infections, among beta-lactam antibiotics, carbapenems or sometimes piperacillin with tazobactam and cefepime remain active, which significantly limits treatment options (Bitsori and Galanakis, 2019). However, *E. coli* isolates that were resistant to carbapenems were not reported in our study. Particularly in the context of UTI but also other infections, rapidly growing resistance to fluoroquinolones is alarming (Bader et al., 2017; Faine et al., 2022). They are mainly drugs of first choice in complicated cases, among others in patients with recurrent UTI or pyelonephritis (European Association of Urology, 2023); what is more, most remain active against ESBL isolates. However, in some countries the widespread use of fluoroquinolones in outpatients has resulted in a large build-up of local resistance. In developing countries, it is up to 85.5%, in Europe the resistance varies between 10.5–39.8% of diagnosed infections, in the USA 5.1–12.1% (Kot, 2019; WHO Regional Office for Europe/European Centre for Disease Prevention and Control, 2022). Our results confirmed that resistance to fluoroquinolones in Poland is at the upper limit. The use of fluoroquinolones has long been controversial due to the observed decreased sensitivity to beta-lactam antibiotics, they can modulate bacterial virulence by interacting with DNA, enhance biofilm formation and they are genotoxic to host mitochondrial DNA (Hangas et al., 2018; Adamus-Białek et al., 2019). The high resistance of *E. coli* presented in this study complies with other authors, who observe that MDR, XDR (extensively drug-resistant) or PDR (pan-drug-resistant) *E. coli* isolates are reported more and more often (Paitan, 2018). This encourages the coordination of global antibiotic protection programs in order not only to implement the best possible solutions, but also to search for new therapeutic options.

In our study, we observed that epidemiologically the most dangerous group seems to be sputum-derived isolates, and surprisingly they differ significantly from LTR-derived isolates, showing the greatest drug resistance to all antibiotics (Figure 4). The sputum isolates come from shallower respiratory tracts, and LRT are mainly isolates derived from bronchial secretions, bronchial lavage and bronchoalveolar lavage. We expect that the difference between sputum and LRT may be related to the appearance of bacteria colonizing the digestive system in the sputum (e.g., because of reflux) or result from contact with prostheses/orthodontic appliances on which a diverse bacterial biofilm is formed. There are also reports of pneumonia not associated with the use of a ventilator appearing because of insufficient oral hygiene in hospitalized patients (Rathbun et al., 2022). However, *E. coli* is a rare respiratory pathogen (Edwards et al., 2019; Schneer et al., 2020). According to Schneer et al. (2020) most of these infections occur in hospitalized patients, and the authors emphasize the link between prolonged hospitalization time and the isolation of resistant *E. coli*. It should be also added that the sputum-derived isolates differ from the urinary isolates, and from the others as well in terms of decrease of the average virulence score of VAGs. The strong negative correlation between virulence potential and resistance confirms their clinical evolution stimulated by antibiotics (Beceiro et al., 2013; Simner et al., 2018; Adamus-Białek et al., 2019; Cepas and Soto, 2020). The presence of sputum-derived *E. coli* in the air bioaerosol is very high due to their colonization of the upper respiratory tract. Owing to their very high resistance and virulence potential, there is a strong justification to monitor them and perhaps develop more effective methods to limit their spread in hospitals.

### 4.4 CRISPR-*Cas* occurrence in correlation with other *Escherichia coli* properties

In recent years, much attention has been focused on CRISPR-*Cas* regions, mostly in the direction of use in gene editing technology. However, we wanted to check whether and how these regions correlate with the bacterial fitness. The best-known purpose of CRISPR sequence and their associated genes (*cas*) is to protect the bacterial genome against invasive foreign DNA (Samai et al., 2015; Whitaker and Vanderpool, 2016; Cao et al., 2022). Multiple genome screenings of *E. coli* strains demonstrated the presence of 4 basic CRISPR-*Cas* systems (Touchon and Rocha, 2010; Long et al., 2019). CRISPR 1 and CRISPR 2 are functionally related, and they are more common in *E. coli* belonging to phylogenetic group A; CRISPR 3 is present in all *E. coli* types; CRISPR 4 is specific for phylogenetic group B. These results are mostly consistent with our observations. Indeed, CRISPR 1 and CRISPR 2 were the most common within group A. The CRISPR 2 was generally most common among our isolates, regardless of their origin. All studied CRISPR were identified in all clinical groups of isolates. In contrast, CRISPR 4 was most specific for group D, as opposed to B. Against the background of CRISPR possession, group B2 stands out the most (Long et al., 2019; Alonso et al., 2021). In the future, it is possible that CRISPR-*Cas* may also constitute markers for the phylogenetic affiliation of group B2. Within this group, the least frequent was CRISPR 1, which is also confirmed by Touchon et al. (2011). The incidence of CRISPR 2, 3 and 4 in group B2 are comparable. The simultaneous presence of CRISPR 4 and the absence of CRISPR 1 was not confirmed among our bacterial isolates (Touchon and Rocha, 2010). However, in the case of phylogenetic group B2, indeed the vast majority of isolates with CRISPR 4 did not have CRISPR 1.

García-Gutiérrez et al. (2015) showed a dependency between the increased number of virulence genes and the reduced number of repeated sequences in *E. coli* CRISPR loci. Moreover, it was shown that pathogenic *E. coli* isolated from a distant ecological niche were varied in terms of number of CRISPR repeats. The correlation between the CRISPR-*Cas* regions and the drug-resistance in *E. coli* was also observed (Heidelberg et al., 2009; Dang et al., 2013; Kim and Lee, 2022). We confirmed that specific types of CRISPR correlate with the resistance to particular antibiotics and virulence-associated genes occurrence (Table 6; Supplementary Table S4). We found a clear correlation between higher prevalence of CRISPR 4, higher drug resistance (except penicillin), and lower virulence potential (except *fimG/H*). In the case of CRISPR 2, lower drug resistance and virulence potential was observed. It may be related to the phenomenon observed by Touchon and Rocha (2010). CRISPR 1 and 2 mainly target phages and CRISPR 3 and 4—plasmids. The mechanism of phage integration into bacterial genomic DNA will correlate with many spontaneous DNA rearrangements, whereas the plasmid-targeting regions of CRISPR seem to be more associated with plasmid drug-resistance mechanisms. There emerges the question: “What came first?” Do antibiotics have an impact on CRISPR-*Cas* stability/variability? Or is it the opposite—does CRISPR-*Cas* influence the bacterial drug resistance and virulence potential? Shehreen et al. (2019) indicate that the role of CRISPR-*Cas* and anti-CRISPRs in the spread of antibiotic resistance is likely to be very different among pathogenic species and clinical environments. The literature on the subject underlines that the multiplex nature of the CRISPR-*Cas* mechanism enables recognition of multiple loci simultaneously, and it leads to large deletions, inversions or translocations (Xiao et al., 2013; Blasco et al., 2014; Essletzbichler et al., 2014; Lin et al., 2014; Liu et al., 2014; Kim and Lee, 2022). The stability of CRISPR-*Cas* in bacterial genome and its mechanism of regulation seems to be crucial in the process of adaptation to unfavorable environmental conditions. An explanation of these phenomena is valuable for a better understanding of the molecular biology of *E. coli*.

To sum up, the understanding of complexed mechanisms of pathogenicity and antibiotic resistance with CRISPR-*Cas* mechanism background is an opportunity to develop better therapies against *E. coli* infections. A closer look at the mechanisms of operation of CRISPR-*Cas* sequences that are closely related to bacteriophages may contribute to further research into new therapeutic options with the use of bacteriophages (Pawluk et al., 2018; Hampton et al., 2020; Kiga et al., 2020). A detailed view at *E. coli* isolated from UTIs in relation to other ExPEC provide material for further consideration. The studied VAGs are probably not specific for UPEC, so perhaps other VAGs and their expression levels under different conditions should be investigated. However, we can indicate clear differences between *E. coli* isolated from different clinical materials—different profiles of virulence potential, resistance, phylogenetic origin and CRISPR regions.

## 5 Conclusion

*E. coli* is a common species in all infections of hospitalized patients. It is also most often isolated from abdominal fluids and urine. Female-correlated *E. coli* infections are more often for UTI, while male-correlated *E. coli* infections are more often for respiratory tract, abdominal fluids, perianal pus and the group of other sources. This observation may be related to the anatomical structure and a more hygienic lifestyle in women.

The concerningly high drug resistance of *E. coli* was recognized among sputum-derived isolates, which may be related to nosocomial infection with multidrug-resistant isolates. Also, penicillins have not been effective in treating more than half of the studied isolates, which is alarming due to their widespread use. Furthermore, we would like to emphasize the questionable use of fluoroquinolones considering the high resistance among *E. coli* and their far-reaching harmful effects.

The presence of studied VAGs was common among all studied *E. coli* isolates, which probably excludes their “urospecificity,” but this would need to be verified in experimental studies. The virulence potential was negatively correlated with antibiotic resistance and positive correlated with membership to phylogenetic group B2, regardless of the origin of the *E. coli* isolates. This confirms that UPEC and other ExPECs are closely related.

The presence of pathogenic factors does not always have to be associated with a more severe course of the disease, but their presence certainly increases such a risk. Monitoring of isolates with a high pathogenic potential is justified.

The antibiotic resistance and virulence potential were negatively correlated with CRISPR 2 and positively correlated with CRISPR 4, which can be related with the DNA metabolism—plasmids or phages integration. Research on CRISPR-*Cas* may provide important evidence about the mechanisms of bacterial pathogenicity and drug resistance.

## Data availability statement

The original contributions presented in the study are included in the article/Supplementary material, further inquiries can be directed to the corresponding author.

## Ethics statement

The studies involving humans were approved by Resolution No. 23/2017 of September 11, 2017, CM UJK. The studies were conducted in accordance with the local legislation and institutional requirements. The human samples used in this study were acquired from a by-product of routine care or industry. Written informed consent for participation was not required from the participants or the participants’ legal guardians/next of kin in accordance with the national legislation and institutional requirements.

## Author contributions

AD: Conceptualization, Data curation, Formal analysis, Investigation, Methodology, Supervision, Validation, Visualization, Writing – original draft, Writing – review & editing. SD: Data curation, Investigation, Resources, Writing – review & editing. AS: Investigation, Resources, Writing – review & editing. MW-K: Formal analysis, Investigation, Validation, Visualization, Writing – review & editing. KZ: Investigation, Methodology, Validation, Writing – review & editing. JB: Investigation, Methodology, Validation, Writing – review & editing. NZ: Investigation, Validation, Visualization, Writing – review & editing. WK: Validation, Visualization, Writing – review & editing. JM: Validation, Visualization, Writing – review & editing. SG: Conceptualization, Data curation, Formal analysis, Funding acquisition, Investigation, Methodology, Project administration, Supervision, Validation, Visualization, Writing – review & editing. WA-B: Conceptualization, Data curation, Formal analysis, Investigation, Methodology, Supervision, Validation, Visualization, Writing – original draft, Writing – review & editing, Funding acquisition.
