# Supplementary material for: Comparative study of virulence potential, phylogenetic origin, CRISPR-Cas regions and drug resistance of Escherichia coli isolates from urine and other clinical materials
Source: Front Microbiol. 2023 Nov 29;14:1289683. doi: 10.3389/fmicb.2023.1289683 (PMC10716328; doi:10.3389/fmicb.2023.1289683)
Supplement: Supplementary file 1 [file Table_1.DOCX]

Supplementary Material

# Supplementary Tables

**Table S1** Occurrence of *E. coli* in clinical materials; negative samples – number of samples where no clinically important bacteria were detected, positive samples – number of samples where clinically important bacteria were detected, including *E. coli*, *E. coli* - number of samples with detected presence of *E. coli*, % - percentage of *E. coli* in the group of positive samples, 2017 – 2022 years of epidemiological data

| Clinical material | diagnostic result | 2017 | 2018 | 2019 | 2020 | 2021 | 2022 |
| --- | --- | --- | --- | --- | --- | --- | --- |
| Urine | negative samples | 4129 | 3778 | 3728 | 4875 | 3485 | 3389 |
|  | positive samples | 1586 | 1558 | 1610 | 1494 | 1883 | 1727 |
|  | *E. coli* | 765 | 643 | 713 | 614 | 715 | 660 |
|  | % | 48.23 | 41.27 | 44.29 | 41.10 | 37.97 | 38.22 |
| Blood | negative samples | 5512 | 6483 | 6804 | 6707 | 6637 | 1377 |
|  | positive samples | 1196 | 1376 | 1210 | 1456 | 1765 | 1574 |
|  | *E. coli* | 154 | 152 | 182 | 181 | 197 | 172 |
|  | % | 12.88 | 11.05 | 15.04 | 12.43 | 11.16 | 10.93 |
| Lower respiratory tracts | negative samples | 207 | 233 | 316 | 365 | 454 | 360 |
|  | positive samples | 1486 | 1450 | 1477 | 1267 | 978 | 1090 |
|  | *E. coli* | 74 | 100 | 91 | 97 | 75 | 86 |
|  | % | 4.98 | 6.90 | 6.16 | 7.66 | 7.67 | 7.89 |
| Sputum | negative samples | 325 | 272 | 286 | 194 | 197 | 164 |
|  | positive samples | 571 | 537 | 564 | 680 | 659 | 504 |
|  | *E. coli* | 24 | 28 | 20 | 13 | 21 | 19 |
|  | % | 4.20 | 5.21 | 3.55 | 1.91 | 3.19 | 3.77 |
| Bedsores and ulcers | negative samples | 18 | 13 | 30 | 21 | 20 | 12 |
|  | positive samples | 240 | 215 | 266 | 229 | 263 | 247 |
|  | *E. coli* | 30 | 17 | 28 | 14 | 22 | 29 |
|  | % | 12.50 | 7.91 | 10.53 | 6.11 | 8.37 | 11.74 |
| Body fluids | negative samples | 259 | 266 | 282 | 227 | 250 | 295 |
|  | positive samples | 229 | 209 | 226 | 169 | 172 | 249 |
|  | *E. coli* | 134 | 106 | 123 | 97 | 117 | 92 |
|  | % | 58.52 | 50.72 | 54.42 | 57.40 | 68.02 | 36.95 |
| Wounds and pusses | negative samples | 451 | 508 | 535 | 380 | 516 | 305 |
|  | positive samples | 1999 | 1784 | 1603 | 1214 | 1488 | 1182 |
|  | *E. coli* | 202 | 245 | 237 | 150 | 165 | 268 |
|  | % | 10.11 | 13.73 | 14.78 | 12.36 | 11.09 | 22.67 |
| Reproductive tracts | negative samples | 259 | 9 | 282 | 227 | 250 | 296 |
|  | positive samples | 212 | 237 | 556 | 590 | 596 | 258 |
|  | *E. coli* | 208 | 182 | 173 | 58 | 48 | 59 |
|  | % | 98.11 | 76.79 | 31.12 | 9.83 | 8.05 | 22.87 |

|  |  | CLINICAL MATERIALS | | | | | | | | | |  |
| --- | --- | --- | --- | --- | --- | --- | --- | --- | --- | --- | --- | --- |
|  | Feature | Blood | LRT | Sputum | FRT | Wound | POW | BF | Perianal pus | Other pus | Other sources | All |
| URINE | *papC* | - | - | U↑ | U↑ | - | - | U↑ | - | - | U↑ | U↑ |
|  | *sfaD/E* | U↑ | - | U↑ | - | - | - | U↑ | - | - | U↑ | U↑ |
|  | *cnf1* | U↑ | - | U↑ | - | - | U↑ | U↑ | - | - | U↑ | U↑ |
|  | *usp* | U↑ | - | U↑ | - | - | - | U↑ | - | - | U↑ | U↑ |
|  | *hlyA* | U↑ | - | U↑ | - | - | - | U↑ | - | - | U↑ | U↑ |
|  | *fimG/H* | - | - | - | - | - | - |  | - | - | - | - |
|  | A | - | - | - | - | - | - | - | - | - | - | - |
|  | B1 | - | - | - | - | U↓ | - | U↓ | - | - | - | - |
|  | B2 | - | - | - | - | U↑ | U↑ | U↑ | - | - | - | - |
|  | C | - | - | - | - | - | - | - | - | - | - | - |
|  | D | - | - | - | - | - | - | - | - | - | - | - |
|  | E | - | - | - | - | - | U↓ | - | - | - | - | - |
|  | F | - | - | - | - | - | U↓ | - | - | - | - | - |
|  | S | U↑ | - | U↑ | - | - | - | U↓ | - | - | - | - |
|  | R_P_ | U↓ | - | - | - | - | - | U↑ | - | - | - | - |
|  | R_C_ | - | U↓ | U↓ | U↓ | U↓ | U↓ | - | - | U↓ | U↓ | U↓ |
|  | R_A_ | - | - | U↓ | - | - | - | U↑ | - | - | - | - |
|  | R_F_ | - | - | U↓ | U↑ | - | U↓ | U↑ | - | - | - | - |
|  | ESBL | - | - | U↓ | - | - | - | U↑ | - | - | - | - |
|  | MDR | - | - | U↓ | - | - | - | U↑ | - | - | - | - |
|  | C1 | - | - | - | - | U↓ | U↓ | U↓ | - | - | - | - |
|  | C2 | - | U↑ | U↑ | - | - | - | - | - | - | - | - |
|  | C3 | - | - | - | U↓ | - | - | - | - | - | - | - |
|  | C4 | - | - | - | - | - | - | - | - | - | - | - |

**Table S2** The correlation between urine-derived and other *E. coli* isolates in relation to a given features. Legend: LTR – lower respiratory tracts; FTR – female reproductive tracts; BF – body fluids; POW – postoperative wound; Other pus (pus-derived isolates that could not be assigned to the created groups, e.g. pus from the ear); Other sources (isolates from sources that could not be assigned to the created groups, e.g. from the throat); All – all other than urine materials; *papC, sfaD/E, cnf1, usp, hlyA, fimG/H* – virulence-associated genes; A, B1, B2, C, D, E, F – phylogenetic groups; S – isolates sensitive for all antibiotics; R_P_ – isolates resistant to at least one antibiotic from penicillins; R_C_ – isolates resistant to at least one antibiotic from cephalosporins; R_A_ – isolates resistant to at least one aminoglycoside; R_F_ - isolates resistant to at least one fluoroquinolone; ESBL – isolates producing ESBL; MDR – isolates resistant to at least one antibiotic from every of three groups (III/IV generations of cephalosporins, aminoglycosides, fluoroquinolones); C1, C2, C3, C4 - CRISPR1, CRISPR2, CRISPR3, CRISPR4; U↑ or U↓ - feature was significantly more often found / less often found in urine-derived isolates; - no statistical significance; chi-square (χ2) test, p<0.05 was considered statistically significant

|  | Features | % | Blood | Urine |
| --- | --- | --- | --- | --- |
| UROSEPSIS | *papC* | **60.7** | - | - |
|  | *sfaD/E* | **32.1** | - | - |
|  | *cnf1* | **25.0** | - | US↓ |
|  | *usp* | **35.7** | - | - |
|  | *hlyA* | **25.0** | - | - |
|  | *fimG/H* | **100.0** | - | - |
|  | A | **0.0** | - | - |
|  | B1 | **10.7** | - | - |
|  | B2 | **75.0** | - | - |
|  | C | **3.6** | - | - |
|  | D | **3.6** | - | - |
|  | E | **7.1** | - | - |
|  | F | **10.7** | - | - |
|  | S | **3.6** | - | US↓ |
|  | R_P_ | **89.3** | US↓ | US↑ |
|  | R_C_ | **17.9** | - | - |
|  | R_A_ | **10.7** | - | - |
|  | R_F_ | **21.4** | - | - |
|  | ESBL | **10.7** | - | - |
|  | MDR | **7.1** | - | - |
|  | C1 | **28.6** | - | - |
|  | C2 | **82.1** | - | - |
|  | C3 | **50.0** | - | - |
|  | C4 | **53.6** | - | - |

**Table S3** The correlation between urosepsis and blood-derived and urine-derived *E. coli* isolates in relation to a given features. Legend: % - percent of uroseptic isolates with given feature; *papC, sfaD/E, cnf1, usp, hlyA, fimG/H* – virulence-associated genes; A, B1, B2, C, D, E, F – phylogenetic groups; S – isolates sensitive for all antibiotics; R_P_ – isolates resistant to at least one antibiotic from penicillins; R_C_ – isolates resistant to at least one antibiotic from cephalosporins; R_A_ – isolates resistant to at least one aminoglycoside; R_F_ – isolates resistant to at least one fluoroquinolone; ESBL – isolates producing ESBL; MDR – isolates resistant to at least one antibiotic from every of three groups (III/IV generations of cephalosporins, aminoglycosides, fluoroquinolones); C1, C2, C3, C4 - CRISPR1, CRISPR2, CRISPR3, CRISPR4; US↑ or US↓ - feature was significantly more often found / less often found in urosepsis isolates; - no statistical significance; chi-square (χ2) test, p<0.05 was considered statistically significant

|  | *papC* | *sfaD/E* | *cnf1* | *usp* | *fimG/H* | *hlyA* | A | B1 | B2 | C | D | E | F | C1 | C2 | C3 | C4 |
| --- | --- | --- | --- | --- | --- | --- | --- | --- | --- | --- | --- | --- | --- | --- | --- | --- | --- |
| Penicillins | - | ↓R | ↓R | ↓R | - | ↓R | - | - | - | - | - | - | - | - | - | - | - |
| Cephalosporins | - | ↓R | - | ↓R | - | ↓R | - | ↓R | - | - | - | - | - | - | ↓R | ↑R | ↑R |
| Aminoglycosides | - | ↓R | - | ↓R | - | - | - | ↓R | ↑R | - | - | - | - | ↓R | ↓R | - | ↑R |
| Fluoroquinolones | ↓R | ↓R | ↓R | ↓R | - | ↓R | - | ↑R | ↓R | - | - | - | ↑R | ↑R | ↓R | ↓R | ↑R |

**Table S4** The correlation between the resistance to the antibiotic groups and other features of *E. coli* isolates. Legend: *papC, sfaD/E, cnf1, usp, fimG/H, hlyA* – virulence-associated genes; A, B1, B2, C, D, E, F – phylogenetic groups; C1, C2, C3, C4 - CRISPR1, CRISPR2, CRISPR3, CRISPR4; R↑ or R↓ feature was significantly more often found / less often found in resistant isolates; - not statistically significant; chi-square (χ2), p<0.05 was considered statistically significan
